# Synergies between Surface Microstructuring and Molecular Nanopatterning for Controlling Cell Populations on Polymeric Biointerfaces

**DOI:** 10.3390/polym12030655

**Published:** 2020-03-13

**Authors:** Andrés Díaz Lantada, Ravi Kumar, Markus Guttmann, Markus Wissmann, Marc Schneider, Matthias Worgull, Stefan Hengsbach, Florian Rupp, Klaus Bade, Michael Hirtz, Sylwia Sekula-Neuner

**Affiliations:** 1 Product Development Laboratory, Mechanical Engineering Department, Universidad Politécnica de Madrid; C/José Gutiérrez Abascal 2, 28006 Madrid, Spain; 2 Institute of Nanotechnology (INT), Karlsruhe Institute of Technology (KIT), Hermann-von-Helmholtz Platz 1, 76344 Eggenstein-Leopoldshafen, Germany; ravi.kumar@kit.edu (R.K.); michael.hirtz@kit.edu (M.H.); 3 Institute of Microstructure Technology (IMT), Karlsruhe Institute of Technology (KIT), Hermann-von-Helmholtz Platz 1, 76344 Eggenstein-Leopoldshafen, Germany; markus.guttmann@kit.edu (M.G.); markus.wissmann@kit.edu (M.W.); marc.schneider2@kit.edu (M.S.); matthias.worgull@kit.edu (M.W.); stefan.hengsbach@kit.edu (S.H.); florian.rupp@kit.edu (F.R.); klaus.bade@kit.edu (K.B.); 4 N.Able GmbH, Hermann-von-Helmholtz Platz 1, 76344 Eggenstein-Leopoldshafen, Germany; ssn@n-able-innovation.com

**Keywords:** polymer microfabrication, biointerfaces, surface microstructuring, direct laser writing, electroplating, hot embossing, molecular nanopatterning, organ-on-a-chip, lab-on-a-chip

## Abstract

Polymeric biointerfaces are already being used extensively in a wide set of biomedical devices and systems. The possibility of controlling cell populations on biointerfaces may be essential for connecting biological systems to synthetic materials and for researching relevant interactions between life and matter. In this study, we present and analyze synergies between an innovative approach for surface microstructuring and a molecular nanopatterning procedure of recent development. The combined set of techniques used may be instrumental for the development of a new generation of functional polymeric biointerfaces. Eukaryotic cell cultures placed upon the biointerfaces developed, both before and after molecular patterning, help to validate the proposal and to discuss the synergies between the surface microstructuring and molecular nanopatterning techniques described in the study. Their potential role in the production of versatile polymeric biointerfaces for lab- and organ-on-a-chip biodevices and towards more complex and biomimetic co-culture systems and cell cultivation set-ups are also examined.

## 1. Introduction

The possibility of controlling cell populations on biointerfaces has recently been put forward as a fundamental key within the “biologization of materials research” strategy, in order to link biological systems with man-made materials and to investigate the interaction between life and matter, in parallel to the development of biomedical and biotechnological applications [1]. In paper [1], Niemeyer and colleagues also highlight the role of biointerfaces that are capable of precisely interacting with cells as fundamental elements, in a global plan towards modular biohybrid systems that may be used as “biofactories of the future” for more sustainable and efficient production processes.

Apart from their promising future, polymeric biointerfaces are already being used extensively in a wide set of biomedical devices and systems, in which controlling cells and cell cultures, for studying or promoting desired cell–cell, cell–material and cell–drug interactions, in conditions as biomimetic and physiological as possible, is necessary. These applications include: cell culture devices, labs- and organs-on-chips, varied microfluidic systems, tissue engineering scaffolds, active devices and even implants with improved biological performance [2,3,4].

Among the different micro and nanomanufacturing processes that can be employed for the generation of biointerfaces capable of interacting at a cellular level and with the ability of controlling cell populations and thereby driving their behavior, dynamics, gene expression, differentiation or fate, it is important to mention the following: techniques derived from the electronic industry, such as photolithography, which allow for the generation of selective patterns using light polymerizing photoresins through masks [5], can be employed for creating controlled surface topographies in a wide set of materials. The controlled topographies can be achieved either by patterning the photoresins or by subsequent chemical etchings of the substrates, upon which the pattern of photoresin is created. Such patterns or textures can be transferred to softer, biomimetic and biologically adequate materials (i.e., PDMS or poly dimethyl siloxane) by using soft-lithography related processes [6].

The mass production of microtextured biodevices with polymeric biointerfaces for studying cell-material interactions can be also achieved by linking rapid prototyping and rapid tooling with microinjection molding [7]. Hot embossing or compression molding provides a remarkable alternative for a wide set of biological and biomedical micro electromechanical systems (bioMEMS) [8]. Electrospinning is not only capable of creating ultrathin fibers for functionalizing 2D surfaces and enhancing their viability as probes for cell culture, but can also be applied to the complex geometries of 3D biodevices, such as tissue engineering scaffolds [9]. Micromolded electrospun hydrogel mats have been also employed recently for creating multiscale constructs, with micro and nanometric features, for interacting with cells and tissues [10]. 

Processes enabling micro and nanopatterning of biomolecules, as functionalizations upon materials surfaces, provide improved ways of fixing/anchoring cells upon surfaces for studying a wide set of cell-material interactions. Among these, dip-pen nanolithography is a very singular nanofabrication technology that can directly write a variety of molecular patterns upon surfaces with excellent resolution, as recently reviewed [11]. Parallel dip-pen nanolithography constitutes an evolution capable of integrating functional biomolecules on subcellular length scales, as a consequence of its constructive nature, high resolution and high throughput [12].

Different techniques of recent development, enabling the creation of design-controlled, super-hydrophilic and super-hydrophobic micropatterns and transitions upon surfaces [13], are promoting many innovative applications in the biomedical and biotechnological fields, especially in connection with high-throughput screening [14,15]. Additive manufacturing of 2D½ bioMEMS and of 3D microstructures upon surfaces are also promoting the almost free-form fabrication of biointerfaces in a wide set of materials, in many cases polymers. Multi-scale combinations of additive manufacturing technologies (AMT) have been reported [16], as well as 3D written structures capable of interacting at the single-cell level [17]. Functionally graded microporous scaffolds upon surfaces, manufactured with biodegradable polymers combining additive processes with surface polymerizations, as biointerfaces for controlled cell cultures have been described [18].

In spite of the varied processes available, in the creation of successful polymeric biointerfaces, researching synergies between them can prove a promising strategy for increasing the level of productivity, throughput and complexity of the biointerfaces created, as required for interacting with the complex human physiologies and for systematically studying cellular mechanisms in disease and cell–material interactions in biomedical devices. 

In this study we present and analyze synergies between an innovative approach for surface microstructuring, aimed at the mass production of polymeric biointerfaces, and a recently developed molecular nanopatterning procedure for the straightforward or “plug-and-play” functionalization of material surfaces. 

Eukaryotic cell cultures upon the biointerfaces developed, both before and after molecular patterning, help to validate the proposal and to discuss the synergies between the surface microstructuring and molecular nanopatterning techniques described. Their potential in the production of versatile polymeric biointerfaces, for being integrated into lab- and organ-on-a-chip biodevices or for being used as components of complex and biomimetic co-culture systems and cell cultivation set-ups, are also examined.

## 2. Materials and Methods 

### 2.1. Computer-Aided Design of Microstructured Surfaces

**Figure 1:**
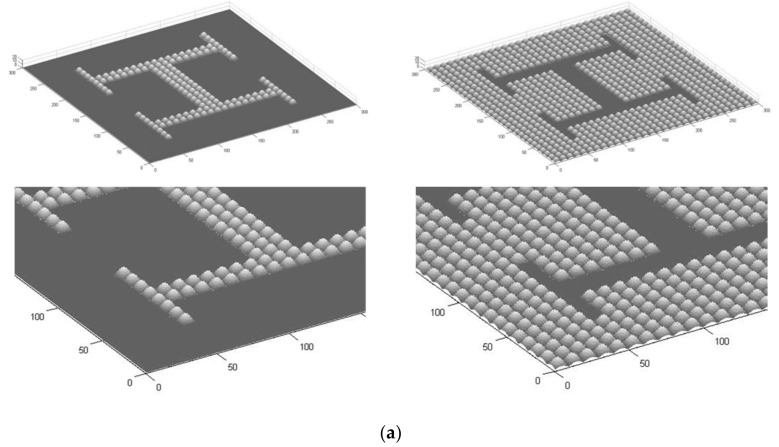

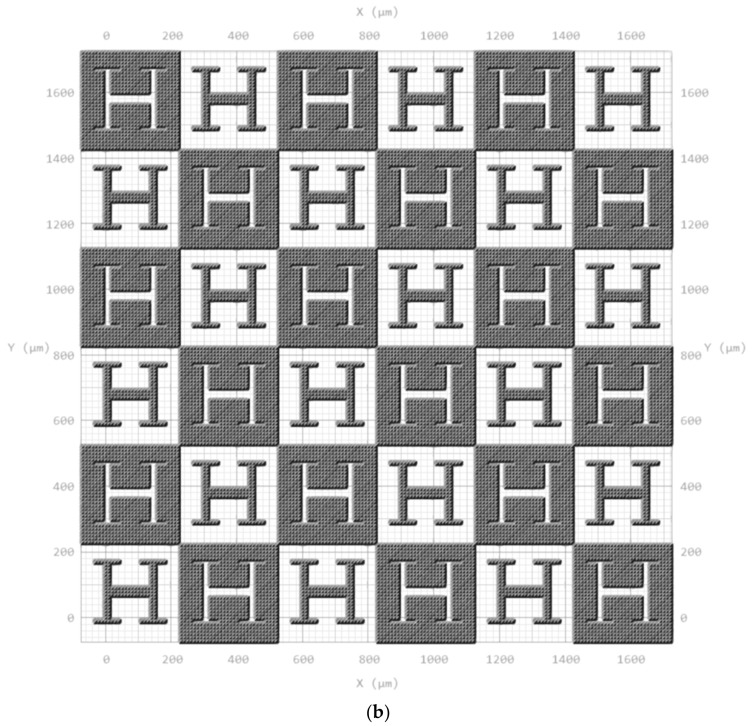
(**a**) Computer-aided design of microstructured surfaces showing both positive and negative alternatives (textured vascular-like region upon a plane and planar vascular-like region surrounded by texture), as well as detailed views of the topographies. (**b**) Design converted into checkerboard-like pattern for higher throughput and more systematic testing ready for direct laser writing.

Multi-scale textures are designed by incorporating them to desired regions of planar surfaces, hence defining design-controlled rough/soft and potentially hydrophobic/hydrophilic transitions, following a previously explained procedure developed by some of the co-authors [19] with minor modifications. The process combines mathematical models for the generation of height matrices further processed with conventional computer-aided design software. In summary, a mathematical equation is evaluated above a grid and defined in accordance with the precision of the subsequent additive manufacturing process. The multi-scale surfaces or textures may be obtained as sum of different micro and nanotopographies. Once generated, they are stored in form of Matlab (The Mathworks Inc, Natick, MA, USA) surfaces-matrices. After generation of the math-based surfaces, the related geometrical information can be processed as [X, Y, Z] matrices and converted into .stl (standard tessellation language) or an interchangeable computer-aided design (CAD) file format, in order to perform additional design operations (i.e., incorporating thickness, applying the surface to a previous geometry, setting some regions to zero, performing Boolean and matrix-based operations, among others). Here we employ, as a mathematical model, the absolute values of a sum of sinusoidal functions, of different amplitude and frequency, as to obtain positive microbumps with a height of 10 mm and occupying regions of 10 × 10 μm^2^. The microbumps have rough surfaces, thanks to the incorporation of smaller bumps of around 2 × 2 × 2 μm^3^, which mimic the morphology of some plant leaves with special surface features and tribological properties [20]. The transition between planar zones and microbumps is aimed at creating differential textures for interacting with cells and controlling cell populations. In this study, we opt for creating patterns imitating microvasculatures, using a checkerboard design pattern (see Figure 1a), in some cases with the microbumps forming multi-branched “H-like” vascular textures, in other cases with the microbumps surrounding planar “H-like” regions.

### 2.2. Production of Microstructured Surfaces

The production of microstructured surfaces is based on a combination of: (1) high-precision additive manufacturing, for the creation of master models; (2) metallization or electroplating, for achieving compression molding inserts or tools; and (3) hot embossing, for the cost-effective production of several replicas employing thermoplastic polymers apt for cell culture applications. In addition, molecular nanopatterning is employed in some experiments, as to analyze the sought synergies between the micro and nano processes under study. The different manufacturing and functionalization procedures, together with the cell culture experiments used for validation purposes, are described in the following subsections. 

#### 2.2.1. Three-Dimensional Direct Laser Writing of Master Models with Design-Controlled Features 

The manufacturing of the master models (directly from the CAD files) is carried out using 3D direct laser writing (3D-DLW) also called 3D laser lithography, a high precision AMT based on two-photon polymerization with ultrashort laser pulses, employing the Photonic Professional System from Nanoscribe GmbH (Kalrsruhe, Germany). In summary, the DLW technology creates objects in a different manner than common 3D printing techniques, which work layer-by-layer. 

Matlab (The Mathworks Inc., Natick, MA, USA) is employed to generate both the layout data and the data input files (in .stl format) that can be used directly by the Nanoscribe conversion software (Figure 1b). The Nanoscribe system uses a laser from Toptica (Femto Fiber pro NIR) with a wavelength of 780 nm. The setup includes a laser combined with an inverted microscope, which is synchronized and controlled by a PC. The beam is guided through an oil-immersion microscope objective (Zeiss, 63X, NA 1.4, Carl Zeiss AG, Oberkochen, Germany,) and focused into a resist (acrylate based Ip-DIP, Nanoscribe), previously placed upon a glass substrate which is rinsed with 2-Propanol. For a better adhesion of the written structures the substrate is heated to 120 °C for 10 min. The mounted glass substrate is moved by motor stages (Physics Instruments M511.HD1, Physik Instrumente GmbH & Co. KG, Karlsruhe, Germany) and a piezoelectric driver (Physics Instruments P-562.3CD, Physik Instrumente GmbH & Co. KG, Karlsruhe, Germany) is used for z-travel. 

In the present study, the structures are created by writing tiles (300 µm × 300 µm for each square of the checkerboard) with the galvo scan unit, which are stitched together to achieve a larger area of 1.8 mm × 1.8 mm. The galvo scan unit scans the laser beam within 150 µm × 150 µm fields. The writing is performed with a slicing distance of 50 nm (z-direction) and with a hatching distance of 75 nm (XY plane). The scan speed of the galvo scan unit is set to 25,000 µm/s. With these parameters and writing strategy, a field of 300 µm × 300 µm can be written in less than 6 min. Development of the structures is performed by washing in propylene glycol methyl ether acetate (PGMEA) two times for 20 min each followed by a third washing step with 2-Propanol for 10 min.

#### 2.2.2. Electroplating of Master Models as Compression Molding Tools 

The microstructured polymeric structures (on the 25 × 25 mm^2^ glass substrate) need to be transferred or transformed into a metallic mold insert or mold cavity by electroforming, following a previously developed process at IMT-KIT (please see authors’ affiliations) with some relevant modifications [21]. First, the glass master with the direct laser written structures is glued into a desired cavity of an 8 mm thick copper substrate. In an evaporation process, the master and substrate get coated with superimposed layers of 7 nm chromium and 40 nm gold. The chromium layer serves as an adhesive layer and the gold layer acts as a conductive plating base. The metallic layers promote a precise galvanic metal deposition throughout the microtextured surfaces. The copper substrate is fixed to a commercial plating holder, which is immersed into a galvanic bath. The nickel electroplating system, working with a boric acid containing (chloride-free) nickel sulphamate electrolyte (T = 52 °C, pH 3.4–3.6), is developed especially for the nickel electroforming of microstructures at IMT-KIT [22]. The use of this electrolyte leads to matt, nearly stress-free, thick nickel layers up to 10 mm without warpage or defects [23].

To promote the exact electroplating of the microtextured surfaces, a progressive growing process is planned: The current density is adjusted to 0.1 A/dm^2^ at the beginning of the plating process and is subsequently increased up to 1.5 A/dm^2^. Electroforming continues until a nickel layer with a thickness of approximately 4 mm is achieved. To support the adhesion of the thick nickel block and to avoid a lift-off during the long plating time (lasting more than 2 weeks), the copper substrate is equipped with six threaded holes for positioning and fixing purposes. 

This electroplating process leads to a very stiff and homogenous metal block with a very uniform thickness, which is necessary for withstanding the mechanical and thermal stresses present in the subsequent hot embossing process. 

The electroplated nickel block with flat surface and without any blowholes and dendrites is finally separated from the copper substrate and mechanically processed (using wire EDM) to the desired external dimensions (32 mm × 32 mm × 2.5 mm), as to fit it into the available hot embossing tool. The DLW glass substrate is removed from the mold insert cavity by using a wet-chemical process and the DLW resist is stripped using a plasma treatment. 

**Figure 2:**
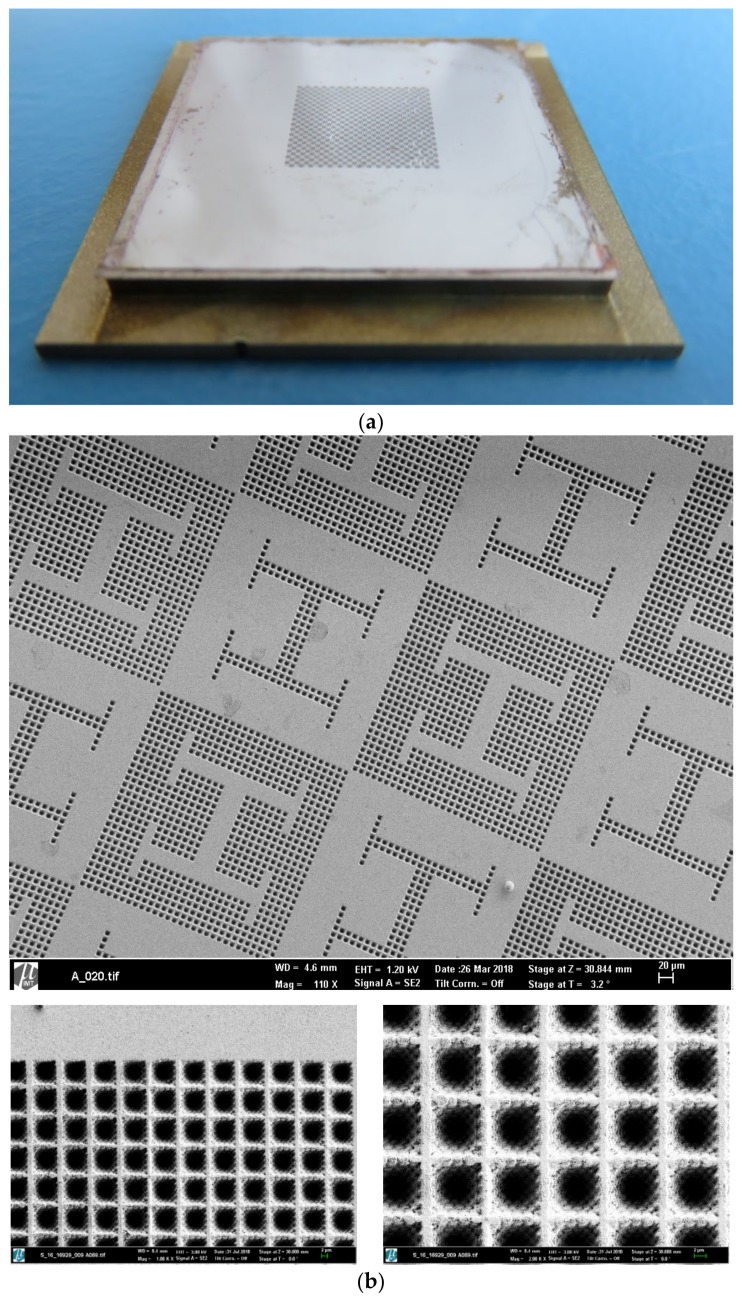
(**a**) Mold insert obtained after electroplating of 3D direct laser written microstructures and employed for hot embossing. (**b**) Details of the mold microstructures with hierarchical geometries, views with different magnification show the multi-scale structures obtained.

A final structure characterization using scanning electron microscopy (SEM, Carl Zeiss AG, Oberkochen, Germany) (see Figure 2 for details) completes the nickel mold fabrication. 

It is important to note that the use of electroforming enables the direct galvanic replication of all structural details (in different scale from nano to micro) of the master microtextures. Moreover, angled side walls and wavy surfaces can also be transferred from the polymeric structures of the master to the metallic mold insert. The mold is then mounted and adjusted into the hot embossing tool for further creation of cell culture polymeric biointerfaces. 

#### 2.2.3. Hot Embossing of Microstructured Surfaces

Polymeric replicas of the vascular-like textured nickel mold are fabricated by the hot embossing of standard poly (methyl methacrylate) (PMMA) foils. PMMA is an interesting thermoplastic material for the biomedical field and adequate for cell culture applications. The polymer foils with a thickness of around 500 µm are placed between mold insert surrounded by a sandblasted steel plate and a polished steel plate, to ensure a smooth back surface of the replicated parts. The hot embossing process is performed by a modified tensile testing machine (Zwick “Retro line”), similar to an embossing system “Jenoptik HEX03” using the following process parameters:
Embossing temperature:165 °CEmbossing force:18 kNDemolding temperature:95 °C

**Figure 3:**
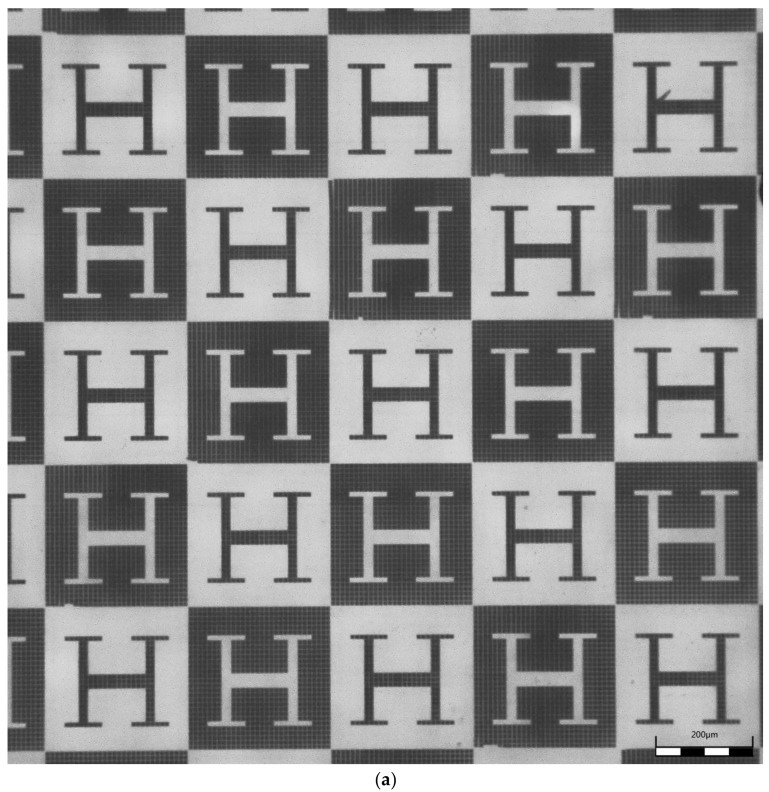

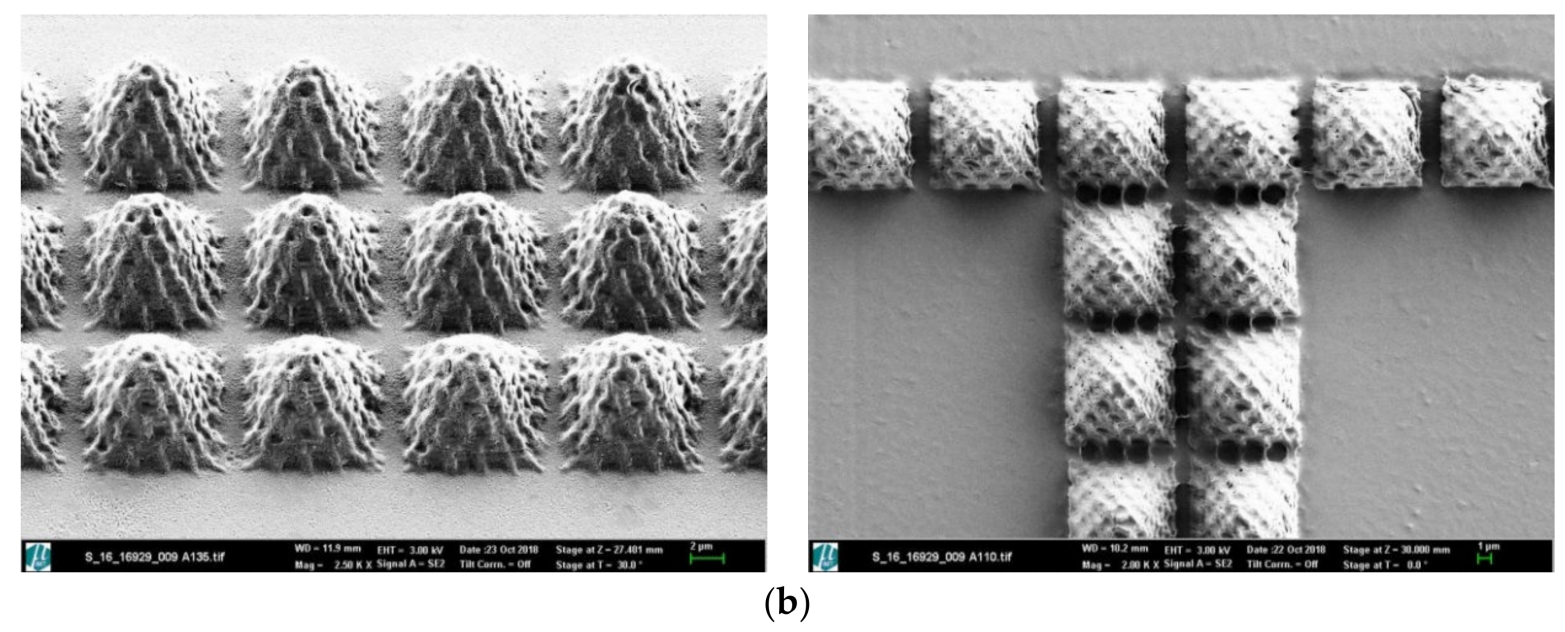
(**a**) Microscopy of hot embossed template after direct laser writing of master models, metallization and pattern transfer to poly (methyl methacrylate) (PMMA) foils. (**b**) Details of hot embossed microstructures showing a remarkable similarity with the original CAD geometries.

The insert details and the structures of the 50 replicated parts in PMMA, illustrated in Figure 2 and Figure 3, show the very detailed dual-scale texture of the microbumps. With this we demonstrate that the hierarchy and the different structure sizes are transferred along the process chain of nickel electroplating and polymer replication by hot embossing with remarkable surface quality.

### 2.3. Cell Culture Experiments

In order to analyze the effects of texture transitions and of molecular functionalizations on cell behavior, 3T3 fibroblasts are employed due to their affinity with fibronectin used as molecular functionalization. Cells are cultured both upon non-functionalized hot embossed PMMA textured samples and upon hot embossed PMMA textured samples, to which molecular patterns are applied as final functionalization to improve cell adhesion, even after phosphate-buffered saline (PBS) washing, (see Section 2.4).

For the first cell culture experiments without molecular functionalization, samples of the microstructured/microtextured surfaces are prepared by washing with 70% ethanol for sterilization and drying with N_2_ for keeping the substrates clean before cell culture. A suspension of 10^6^ cells/mL is prepared and employed for preliminary testing.

**Figure 4:**
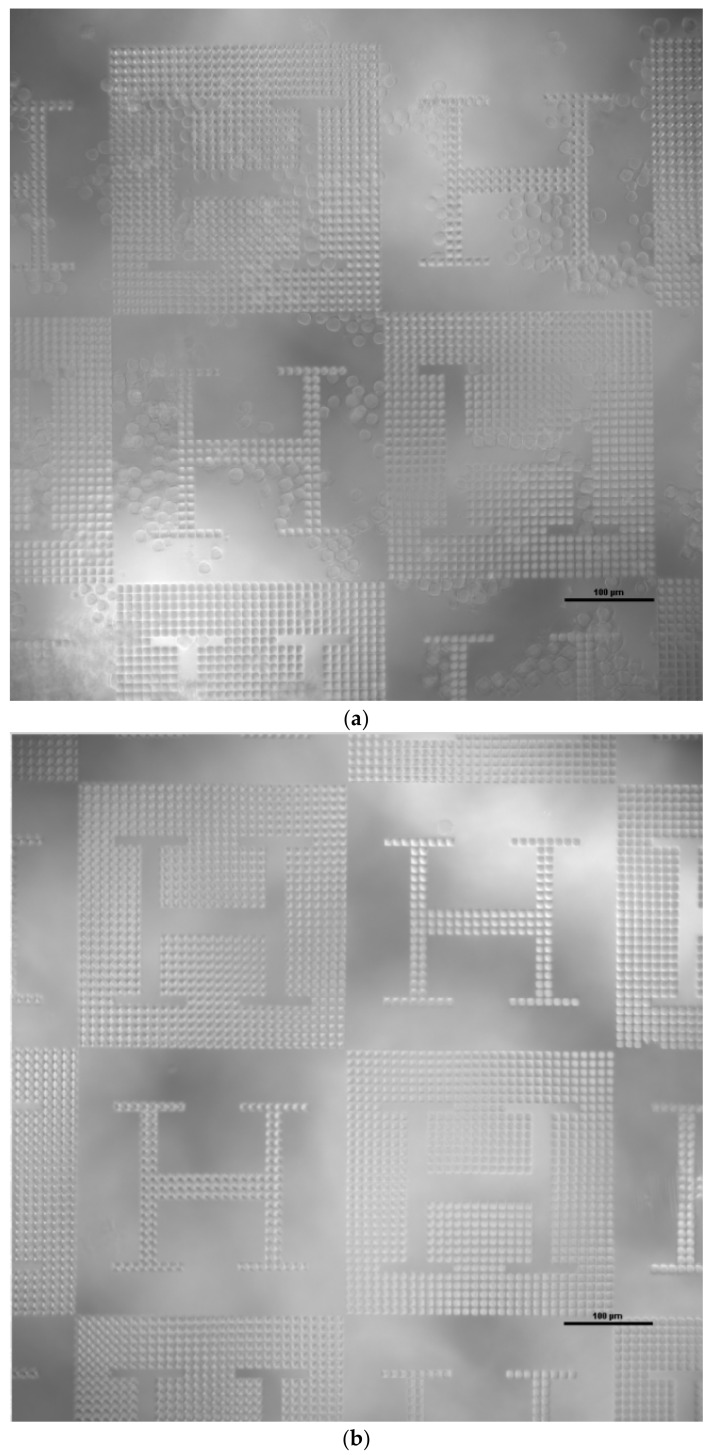
(**a**) 3T3 fibroblast cells after 40 min of culture upon a microtextured sample showing how cells organized due to the presence of topographical transitions. (**b**) Sample after final washing with PBS (almost all cells are removed).

In the first test, the samples are placed in a Petri dish and 5 mL of cell solution is gently deposited upon them, leaving the samples in the incubator for 2 h. In the second test, a sample is placed under the microscope (Eclipse 80i, Nikon Corporation, Tokyo, Japan), 500 μL of cell suspension is deposited upon the sample and living cell imaging is performed for 5 min (Figure 4a). Then the sample is transferred to a warm plate to heat the cells up and image them with 40 min intervals to monitor cell attachment to the substrate (Figure 4b)

For the final cell culture experiment with fibronectin functionalization (as described in Section 2.4), samples of microtextured surfaces are employed, cleaned as previously described and functionalized with fibronectin labelled with CF488 fluorescent dye before culturing the 3T3 cells for 3 h. Fluorescent microscopy is performed to recognize the fibronectin patterns in green and using 4′,6-diamidino-2-phenylindole (DAPI) for staining cell nuclei in blue for cells evaluation. Samples with and without functionalization are also compared.

### 2.4. Molecular Nanopatterning of Microstructured Surfaces

**Figure 5:**
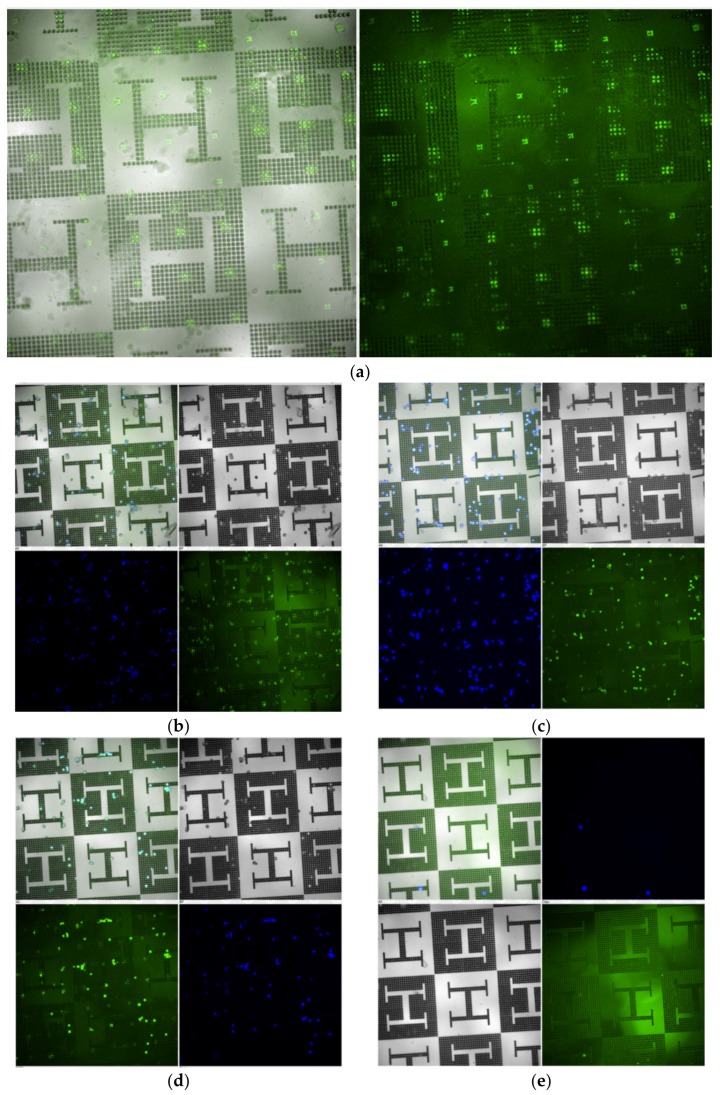
(**a**) Fibronectin functionalization obtained by polymer pen lithography (PPL) shown as a pattern of green dots/squares. (**b**,**c**,**d**) Cell culture results on fibronectin-functionalized PMMA replicas. (**e**) Cell culture results on non-functionalized PMMA replicas. Fluorescent microscopy: DAPI (blue) shows cell nuclei, fibronectin (green) is indicating where the adhesion pattern is printed. The scale bar is 100 µm.

Molecular patterning of fibronectin spots (Figure 5a) is performed by polymer pen lithography (PPL) [24] employing an NLP 2000 system (NanoInk, Inc., Skokie, IL, USA) modified to accommodate a PPL stamp. The system can deposit sub-micron sized features from a wide variety of materials with high location precision by employing high-resolution nanopositioning stages. Using the NLP 2000 system, a matrix of fibronectin labelled with CF488 fluorescent dye spots with spot distances of 100 µm is applied, so that the molecular functionalization acts both upon planar and upon textured regions of the samples. Microcopy is performed to analyze the number of functionalized spots upon planar and upon textured regions for further cell counting and synergies assessment purposes.

## 3. Results

The proposed design and manufacturing processes produced polymeric substrates (thermoplastic PMMA) with microstructured surfaces, as can be observed in Figure 1, Figure 2 and Figure 3. First, Figure 1a presents the computer-aided design of microstructured surfaces as an object of study, showing both positive and negative alternatives, (that is, a textured vascular-like region upon a plane and a planar vascular-like region surrounded by texture), as well as detailed views of the topographies. Figure 1b presents the previous design converted into checkerboard-like pattern, for higher throughput and more systematic testing, ready for direct laser writing.

Figure 2 then presents the mold insert and Figure 3a shows a microscopy of the hot embossed PMMA template or substrate, obtained after the direct laser writing of master models, metallization and pattern transfer by compression molding. Some details of the hot embossed microstructures, which present remarkable similarity with the original CAD geometries, are included in Figure 3b.

In consequence, it is important to highlight that the proposed techniques and processes allow for the creation of microstructures with overall sizes in the order of magnitude of common cell types (e.g., 10 × 10 × 10 μm^3^). The generated microstructures, which mimic the typical multi-scale bumpy features common of the epidermis of different types of plants and leaves, incorporate finer details (in the 2 × 2 × 2 μm^3^ range) with the potential to promote even subcellular interactions. A close look at the “H-like” vascular patterns helps realize that the different branches of the “H” vascular patterns have different widths: the central channels have a width of 30 μm for letting groups of three cells interact, the lateral channels have a width of 20 μm for trapping cells in couples and the more external channels of the “H” figures have a width of 10 μm, within which only a row of single cells can be arranged.

The gradients of width in the template have a twofold purpose: on one hand, changing the thickness systematically allows for the verification of the precision and viability of the manufacturing processes for achieving multi-scale and complex surface topographies, which is confirmed. On the other hand, it may support the topography-guided organization of cells, forming rows or columns, duplets and triplets, for a wide set of potential uses for labs- and organs-on-chips.

Moreover, these positive and negative features, in the form of checkerboard-like pattern, are aimed at having a versatile cell culture platform with 3D microtextures as, depending on the cell type (s) cultured, some cells may adhere to the textures, while some others may be repelled by them.

Ideally, this could lead to viable co-cultures of endothelial cells (for the vascular structures) and of other cell types (for the parenchymal tissues surrounding the vasculatures), in which the positions of the different cells may be controlled by the designed surface textures, as to achieve biomimetic physiological structures on chips.

In addition, by using a couple of microtextured surfaces or chips, culturing two different cell types upon them and placing the chips face-to-face (with the parenchymal regions facing the vascular structures), it may be possible to achieve sandwiched microenvironments to study cell–cell and cell–material interactions in depth [25]. Furthermore, the defined transitions of planar and microtextured regions can be applied to the creation of microfluidic devices “on chips”, in which wet and dry zones may be defined just by design-controlled surface texturing upon a single functional layer or surface. This may support the development of a new generation of labs- and organs-on-chips, in which the number of components (layers, tubes, membranes, clamping elements) may be reduced, once fluids and cells are controlled by surface texturing, for which the employment of additional surface functionalizations, such as molecular nanopatterning to more precisely fix cell positions, will surely be needed, as we complete further analysis.

Once manufacturability is confirmed, cell culture experiments, both upon non-functionalized and functionalized microstructured surfaces, as detailed in Section 2.3 and Section 2.4, lead to interesting results and discussions regarding cell–(material, topography, molecular functionalization) interactions. As first experimental results, Figure 4a presents a set of 3T3 cells, imaged after 40 min of culture upon a microtextured sample, and shows how cells are organized due to the presence of topographical transitions (rows, duplets, triplets and clusters can be observed). Figure 4b shows the same sample, after washing with PBS, which removes almost all cells.

We conclude that, although the use of controlled microtopographies upon PMMA substrates can be applied for organizing cellular suspensions on chips, it is not enough for fixing the cells to desired positions for long-term studies. In order to fix the cells to desired (and design-controlled) positions for longer cell culture studies, in which long-term viability, gene-expression processes, progress of disease or effects of drugs for healing (among other options) can be studied, it is necessary to post-process the hot embossed samples and to convert them into more active biointerfaces. In this study, we resorted to molecular patterning of fibronectin using PPL. Fibronectin is a high-molecular weight glycoprotein of the extracellular matrix that binds to membrane-spanning receptor proteins called integrins, as well as to other extracellular matrix proteins, including collagen, fibrin and heparan sulfate proteoglycans [26].

Details of the functionalization and subsequent cell culture protocols for impact assessment are given in Section 2.3 and Section 2.4. As an example of the post-processing performed to some hot embossed samples, Figure 5a shows the fibronectin functionalization obtained by PPL, highlighted in fluorescent microscopy as a pattern of green dots/squares. Existing as an equally spaced pattern upon a checkerboard design, a similar number of functionalized dots/squares was applied upon the planar regions (the pale gray zones of the checkerboard) and upon the microtextured or microstructured ones (the dark gray zones of the checkerboard).

**Figure 6:**
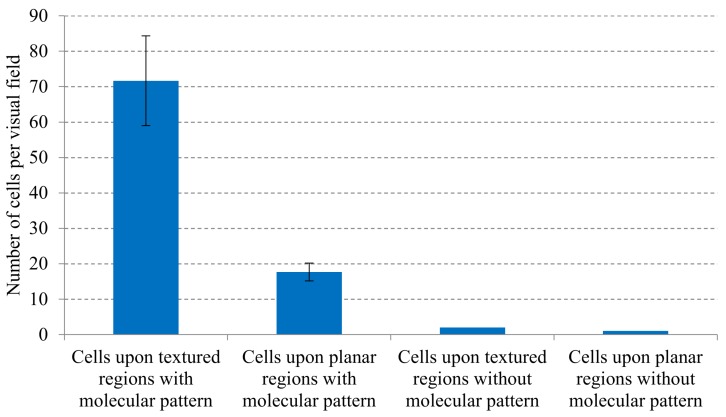
Influence of microtexturing and molecular patterning on the cellular attachment to desired positions of the PMMA substrates. Analysis performed by counting the number of living attached cells per visual field (800 µm × 800 µm area).

The results of the cell culture upon fibronectin-functionalized PMMA replicas can be observed in Figure 5b–d, while cell culture result upon non-functionalized PMMA substrates is presented in Figure 5e, showing just one image as an example, due to the lack of cells. Subsequently, with the support of Figure 5b–d, the influence of microtexturing and molecular patterning on the cellular attachment to desired positions of the PMMA substrates is studied and presented in the summarizing graph of Figure 6. The analysis is performed simply by counting the number of living cells (per visual field) attached to planar and to textured regions of the functionalized and non-functionalized substrates.

According to results presented in Figure 5 and summarized in Figure 6, the microstructured PMMA samples are not capable of fixing the fibroblasts to desired positions, unless a molecular functionalization is applied. Without the functionalization with fibronectin, the topographies do not have any special impact on cell attachment, only on the organization of cells in suspension, as also shown in the first experiment presented in Figure 4. However, once functionalized, an interesting synergy between surface microstructuring and molecular patterning can be observed: cells seem to attach in a much better way to regions, in which microtextures and fibronectin co-exist.

## 4. Discussion: Potentials, Limitations and Continuation Proposals

The main novelty of the presented study, as compared with previous studies from our team and with the introduced state-of-the-art methods, relies on: (1) the degree of multiplexation, achieved by 3D direct laser writing of large fields, in which designs are repeated in a checkerboard pattern; (2) the precision of mass production tools obtained by electroplating and employed for the systematic replication of polymeric test probes by hot embossing; and (3) the combined utilization of surface texturing and molecular nanopatterning for improving the degree of control upon cells on chips. Such synergies are, to our knowledge, presented and analyzed for the first time here.

In previous research [27] fibronectin was found to be appropriate for controlling cell positions upon cell culture plates and devices, but here we show a reinforced ability, which we attribute to the adjuvant effect of the design-defined microstructures. It is likely that the topographies created, which include details in the subcellular scale range, provide anchorage elements for the cells, in which the pseudopodia of the eukaryotic cell membrane can grasp in an enhanced way, as compared to the situation upon completely planar regions. These results are in agreement with varied reported effects of surface topography on cell adhesion and motility, which were described in recent research papers [28,29,30]. Apparently, the microtextures do not only provide anchorage elements for the cells, but may also serve as reservoirs for the patterned fibronectin, which may explain the special synergy found between texturing and molecular patterning. Although in this study the PPL process has been applied to create a periodic pattern of fibronectin spots, innovative modifications to the traditional PPL technique allow for more customizable and flexible surface functionalizations, including the creation of complex paths and of both periodic and aperiodic patterns, even with multiple functionalizing inks, in ways as versatile as those achievable with ink upon paper when using a desktop ink-jet printer, but at the micro and nanoscale [31,32,33,34]. Future studies using more flexible printing methods will progress in these directions.

Regarding Figure 6, cells are counted after fixing and nuclei staining. In consequence, there is no relation between the initial concentration (1 million cells) of living cells and the fixed ones that survive the whole experiment. Unfixed cells are washed away, even if they might have been still searching for anchorage regions. The figure legend denotes that the number of fixed cells per visual field (800 µm × 800 µm square area) is presented. Although we are not able to observe cell division, the distribution of adhered cells over different regions, due to the presence of textures and fibronectin, can be observed clearly.

It is important to consider that cell adhesion involves the interaction of cells with other cells or with the extracellular matrix (ECM), like fibronectin, which is expressed by fibroblasts and many other cells. Hence, fibroblast cells are chosen as the best cell model for attaching to the printed fibronectin pattern. Fibronectin is an important constituent of the extracellular matrix and it interacts with specific domains on the fibroblast surface. Namely, it binds to membrane-spanning receptor proteins-integrins. Integrins are proteins that function mechanically, by attaching the cell cytoskeleton to the extracellular matrix, and biochemically, by sensing whether adhesion has occurred. In any case, since fibronectin is a major component of the extracellular matrix, these observations suggest that it may provide at least one of the signals, by which the matrix conveys the competence that permits fibroblasts to replicate in the presence of an appropriate progression signal, as previous studies also suggest [35,36].

We would like to point out that the technological aspect was developed and demonstrated as a type of pilot production plant, which combines bioinspired design approaches, 3D direct laser writing, electroplating (for the creation of mass-production tools), hot embossing (for creating replicas of polymeric sheets that enable systematic testing) and molecular nanopatterning for improving the degree of control upon cultured cells. The hot embossed polymeric sheets or biointerfaces prove interesting in terms of usability, as compared with thicker injection-molded, especially regarding manipulation and visualization. The presented strategy has been preliminarily validated with a robust cell line, and it was also chosen for its affinity with the fibronectin spots employed as molecular pattern, although we expect to progress towards the application of this set of tools for developing complete organ-on-chips and for answering specific biological questions.

In regards to more relevant industrial applications, the manufacture of technically relevant samples, which may require functional areas of some cm^2^, is still a limitation of presented chain of processes, as the writing field in available two-photon polymerization systems is still limited to just some hundreds of μm^2^. Nevertheless, by stitching writing fields, it is possible to manufacture samples of several mm^2^, at least very adequate for in vitro testing, as has been demonstrated. The attainable accuracy is, in any case, outstanding and remarkable for conceptual proofs of different micro and nanosystems, but the writing speed and attainable part size are still limited for mass production.

Once photopolymerization with micrometric precision improves in terms of achievable part sizes, the mass-production of a wide set of surface microstructured devices and components will be relatively straightforward, by just directly converting polymeric master models into hot embossing tools by electroplating and subsequent compression molding, as shown in this study. Polymer pen lithography also demonstrates precise and versatile molecular functionalizations, as post-process for enhancing the performance of mass-produced polymeric components by hot embossing.

Alternatively, a purposely designed microcontact stamp that matches the layout of the microstructures may provide even more control over the distribution of the functionalization on the positive and negative areas. It may also synergize interestingly with mass-produced substrates by micro-injection molding, as an alternative to hot embossing when three-dimensional shape complexity is needed.

## 5. Conclusions

In our opinion, the combined set of design, manufacturing, microstructuring and surface nano/micro-patterning techniques presented could be instrumental for the development of a new generation of functional polymeric biointerfaces. In the future, if the textures are designed to create hydrophobic/hydrophilic transitions by gradients of topography, it may lead to more straightforward control of cellular populations and cell clusters upon polymeric biointerfaces, complementing relevant processes based on click chemistry [13,14,15] and surface functionalization with polymer nano-brushes [37,38].

The possibility of interacting with cells in suspension by design controlled topographies that define channels and chambers upon polymeric substrates shows promising results towards innovative labs- and organs-on-chips, especially if molecular patterns are synergistically employed for fixing specific cell types to desired positions in cell co-culture systems. This may be translated to the functionalization of polymeric implants also, for the promotion of interesting cell–material interactions and potentially enhanced biocompatibility and bioactivity.

## References

[1] Niemeyer C.M., Bastmeyer M., Bräse S., Lahann J., Woell C. (2018). White Paper on the Biologization of Materials Research. Preprints.

[2] Sun A., Lahann J. (2009). Dynamically switchable biointerfaces. Soft Matter.

[3] Hutmacher D., Chrzanowski W. (2014). Biointerfaces: Where Material Meets Biology.

[4] Khademhosseini A., Langer R., Borenstein J., Vacanti J.P. (2006). Microscale technologies for tissue engineering and biology. Proc. Natl. Acad. Sci. USA.

[5] Xia Y., Whitesides G.M. (1998). Soft lithography. Annu. Rev. Mater. Sci..

[6] Madou M., Wang C., Bhushan B. (2012). Photolithography. Encyclopedia of Nanotechnology.

[7] Díaz Lantada A., Piotter V., Plewa K. (2015). Toward mass production of microtextured microdevices: Linking rapid prototyping with microinjection molding. Int. J. Adv. Manuf. Technol..

[8] Worgull M. (2009). Hot Embossing.

[9] Duque-Sanchez L., Brack N., Postma A., Meagher L., Pigram P.J. (2019). Engineering the biointerface of electrospun 3D scaffolds with functionalized polymer brushes for enhanced cell binding. Biomacromolecules.

[10] Aldana A.A., Malatto L., Rehman M.A.U., Boccaccini A.R., Abraham G.A. (2019). Fabrication of Gelatin Methacrylate (GelMA) Scaffolds with Nano- and Micro-Topographical and Morphological Features. Nanomaterials.

[11] Liu G., Hirtz M., Fuchs H., Zheng Z. (2019). Development of dip-pen nanolithography (DPN) and Its derivatives. Small.

[12] Sekula S., Jeanette F., Susanne W.-R., Peter N., Stefan S. (2008). Multiplexed lipid dip-pen nanolithography on subcellular scales for the templating of functional proteins and cell culture. Small.

[13] Ueda E., Levkin P.A. (2013). Emerging applications of superhydrophilic-superhydrophobic micropatterns. Adv. Mater..

[14] Geyer F.L., Ueda E., Liebel U., Grau N., Levkin P.A. (2011). Superhydrophobic–Superhydrophilic Micropatterning: Towards Genome-on-a-Chip Cell Microarrays. Angew. Chem. Int. Ed..

[15] Ueda E., Geyer F.L., Nedashkivska V., Levkin P.A. (2012). Droplet microarray: Facile formation of arrays of microdroplets and hydrogel micropads for cell screening applications. Lab Chip.

[16] Hengsbach S., Díaz Lantada A. (2014). Rapid prototyping of multi-scale biomedical microdevices by combining additive manufacturing technologies. Biomed. Microdevices.

[17] Hippler M. (2019). 3D Scaffolds to Study Basic Cell Biology. Adv. Mater..

[18] Gunnewiek M.K., Luca A.D., Bollemaat H.Z., van Blitterswijk C.A., Vancso G.J., Moroni L. (2015). Creeping proteins in microporous structures: Polymer brush-assisted fabrication of 3D gradients for tissue engineering. Adv. Healthc. Mater..

[19] DíazLantada A., Hengsbach S., Bade K. (2017). Lotus-on-chip: Computer-aided design and 3D direct laser writing of bioinspired surfaces for controlling the wettability of materials and devices. Bioinspiration Biomim..

[20] Barthlott W., Neinhuis C. (1997). Purity of the sacred lotus, or escape from contamination in biological surfaces. Planta.

[21] Wissmann M., Guttmann M., Hartmann M., Hofmann A., Hummel B. Alternative Mould Insert Fabrication Technology for Micromoulding by Galvanic Replication. Proceedings of the Symposium on Design, Test, Integration and Packaging of MEMS/MOEMS (DTIP 2010).

[22] Schanz G., Bade K., Baltes H. (2005). Microelectroforming of Metal. Advanced Micro and Nanosystems, Vol. 4, Microengineering of Metals and Ceramics.

[23] Guttmann M., Schulz J., Saile V., Baltes H. (2005). Lithographic Fabrication of Mold Inserts. Advanced Micro and Nanosystems, Vol. 3, Microengineering of Metals and Ceramics.

[24] Huo F., Zheng Z., Zheng G., Giam L.R., Zhang H., Mirkin C.A. (2008). Polymer Pen Lithography. Science.

[25] Ballester-Beltran J., Lebourg M., Salmeron-Sanchez M. (2013). Dorsal and ventral stimuli in sandwich-like microenvironments. Effect on cell differentiation. Biotechnol. Bioeng..

[26] Pankov R., Yamada K.M. (2002). Fibronectin at a glance. J. Cell Sci..

[27] Wang Z., Lang B., Qu Y., Li L., Song Z., Wang Z. (2019). Single-cell patterning technology for biological applications. Biomicrofluidics.

[28] Hoffmann M., Kuska J.-P., Zscharnack M., Loeffler M., Galle J. (2011). Spatial organization of mesenchymal stem cells in vitro—Results from a new individual cell-based model with podia. PLoS ONE.

[29] Jachetti E., Di Renzo C., Meucci S., Nocchi F., Beltram F., Cecchini M. (2014). Wharton’s jelly human mesenchymal stem cell contact guidance by noisy nanotopographies. Nat. Sci. Rep..

[30] Díaz Lantada A., Alarcón Iniesta H., García-Ruíz J.P. (2015). Multi-channeled polymeric microsystem for studying the impact of surface topography on cell adhesion and motility. Polymers.

[31] Brinkmann F., Hirtz M., Greiner A.M., Weschenfelder M., Waterkotte B., Bastmeyer M., Fuchs H. (2013). Interdigitated Multicolored Bioink Micropatterns by Multiplexed Polymer Pen Lithography. Small.

[32] Arrabito G., Schroeder H., Schröder K., Filips C., Marggraf U., Dopp C., Venkatachalapathy M., Dehmelt L., Bastiaens P.I.H., Neyer A. (2014). Configuarble Low-Cost Plotter Device for Fabrication of Multi-Color Sub-Cellular Scale Microarrays. Small.

[33] Kumar R., Weigel S., Meyer R., Niemeyer C.M., Fuchs H., Hirtz M. (2016). Multi-color polymer pen lithography for oligonucleotide arrays. Chem. Commun..

[34] Kumar R., Urtizberea A., Ghosh S., Bog U., Rainer Q., Lenhert S., Fuchs H., Hirtz M. (2017). Polymer Pen Lithography with Lipids for Large-Area Gradient Patterns. Langmuir.

[35] Bitterman P.B., Rennard S.I., Adelberg S., Crystal R.G. (1983). Role of fibronectin as a growth factor for fibroblasts. J. Cell Biol..

[36] Lehnert D., Wehrle-Haller B., David C., Weiland U., Ballestrem C., Imhof B.A., Bastmeyer M. (2004). Cell behaviour on micropatterned substrata: Limits of extracellular matrix geometry for spreading and adhesion. J. Cell Sci..

[37] Rodriguez-Emmenegger C. (2013). Controlled cell adhesion on poly(dopamine) interfaces photopatterned with non-fouling brushes. Adv. Mater..

[38] Parrillo V., de Los Santos Pereira A., Riedel T., Rodriguez-Emmenegger C. (2017). Catalyst-free ‘click’ functionalization of polymer brushes preserves antifouling properties enabling detection in blood plasma. Anal. Chim. Acta.

